# Lidar-derived structural-complexity data across four experimental forests

**DOI:** 10.1016/j.dib.2024.110955

**Published:** 2024-09-18

**Authors:** C. Wade Ross, E. Louise Loudermilk, Joseph J. O'Brien, Grant Snitker

**Affiliations:** a Tall Timbers, 13093 Henry Beadel Dr, Tallahassee, FL 32312, United States; b US Forest Service, Southern Research Station, 320 E Green St, Athens, GA 30602, United States; c New Mexico Consortium, 800 Bradbury Dr SE, Suite 213, Albuquerque, NM 87106, United States

**Keywords:** ALS, Canopy height model, Crown delineation, Digital elevation model, Forest structure, Tree detection

## Abstract

Structural complexity refers to the three-dimensional arrangement and variability of both biotic and abiotic components of an ecosystem. Metrics that characterize structural complexity are often used to manage various aspects of ecosystem function, such as light transmittance, wildlife habitat, and biological diversity. Additionally, these metrics aid in evaluating resilience to disturbance events, including hurricanes, bark-beetle outbreaks, and wildfire. Recent advances in wildland fire modelling have facilitated the integration of forest structural complexity metrics into the QUIC-Fire model, enabling real-time prediction of fire spread and behaviour by simulating interactions between fire, weather, topography, and forest structure. While QUIC-Fire is designed to be highly adaptable, model performance depends on the availability and accuracy of local data inputs. Expanding the model's usability across different regions can be facilitated by the availability of more comprehensive and high-quality data. Thus, the primary goal behind the data products we developed was to establish a basis for collaborative research across various disciplines, particularly within the focal areas of the Southern Research Station, such as forestry, wildland fire, hydrology, soil science, and cultural resources at Bent Creek, Coweeta, Escambia, and Hitchiti Experimental Forests (EFs).

Airborne laser scanning (ALS) was used to collect point-cloud data for each EF during the leaf-off season to minimize interference from foliage. Subsequent processing of the raw lidar data involved outlier detection and filtering, ground and non-ground classification, and the computation of a variety of metrics representing various aspects of topography and forest structure at both the pixel-level and the tree-level. Pixel-level topographic data products include: digital elevation model (DEM), slope, aspect, topographic position index (TPI), topographic roughness index (TRI), roughness, and flow direction. Forest structural-complexity metrics include canopy height, foliar height diversity (FHD), vertical distribution ratio (VDR), canopy rugosity, crown relief ratio (CRR), understory complexity index (UCI), vertical complexity index (VCI), canopy cover, mean vegetation height, and the standard deviation of vegetation height. Tree-level data products were computed from the point cloud using multiple algorithms to perform individual tree detection (ITD) and individual tree segmentation (ITS). The datasets have been harmonized and are openly accessible through the USDA Forest Service Research Data Archive.

Specifications Table

**Table:** 

Subject	Forestry
Specific subject area	Forest structural complexity data, tree detections, crown delineations, and topography
Type of data	Vector (GeoPackage), Raster (GeoTiff)
Data collection	Aerial laser scanning was collected in the winter/early spring during the leaf-off period using an aircraft-mounted Optech Galaxy T2000 lidar sensor.
Data source location	Country: United States. Institution: US Forest Service, Southern Research Station. Bent Creek Experimental Forests: 35.050580, −83.450054 Coweeta Experimental Forests: 31.007539, −87.078571 Escambia Experimental Forests: 33.057078, −83.679620 Hitchiti Experimental Forest: 35.484250, −82.633346
Data accessibility	Repository name: US Forest Service Research Data Archive Data identification number: https://doi.org/10.2737/RDS-2024-0019 Direct URL to data: https://www.fs.usda.gov/rds/archive/catalog/RDS-2024-0019 Raw ALS point-cloud data are located at https://app.box.com/s/4s3412g8mtky0hb6wb63a44epv08c22o
Related research article	none.

## 1 Value of the Data

• These data are useful in characterizing and understanding forest structural complexity across differing ecosystems within the southeastern US. These data include information related to elevation, slope, aspect, topographic position index (TPI), topographic roughness index (TRI), roughness, flow direction, canopy height, foliar height diversity (FHD), vertical distribution ratio (VDR), canopy rugosity, crown relief ratio (CRR), understory complexity index (UCI), vertical complexity index (VCI), canopy cover, mean vegetation height, and the standard deviation of vegetation height.
• These data support research across several disciplines, including ecology, forestry, hydrology, soil science, wildland fire science, and cultural resources. By providing detailed physical measurements of forest structure, the data facilitates studies such as habitat suitability, species distribution, environmental change impacts, and fire spread.
• Researchers can utilize the forest structural complexity metrics to deepen their analyses, replicate structural metrics for other forests, and extend their investigations into forest dynamics. Various methodological approaches can be applied to further explore this dataset.
• By offering a detailed snapshot of current forest conditions, the data serve as a baseline for ongoing and future longitudinal studies. Researchers can track changes over time, assess the effectiveness of forest management practices, and study ecological responses to climatic variations.
• These data also provide a valuable resource for educational programs focused on environmental science and forest management. It allows students and trainees to work with real-world data, enhancing their learning experience and preparing them for professional roles in environmental and forestry sciences.

## 2 Background

This dataset [1] was created to support collaborative research efforts across several disciplines, including ecology, forestry, hydrology, soil science, wildland fire science, and cultural resources. The motivation stems from a methodological need to enhance the precision of forest dynamic models and wildland fire models, particularly QUIC-Fire, by incorporating metrics that characterize forest structure, such as canopy height, topography, tree crowns, and bole height [2]. This model requires detailed spatial metrics to accurately simulate forest dynamics and fire behaviour. Metrics such as canopy height, topography, tree crowns, and bole height are crucial for these simulations, as they directly influence model outputs on vegetation growth, species distribution, and fire spread. By integrating such metrics into the models, the dataset aims to enhance the precision of predictions and provide a more nuanced understanding of forest ecosystems and fire dynamics. This enhanced modelling capability is intended to support more effective management practices and policy decisions in forestry and land management. The dataset thereby serves as a valuable resource for researchers looking to apply robust modelling techniques to complex ecological and environmental challenges.

## 3 Data Description

The data are stored in both raster- and vector-based formats and are projected to the Universal Transverse Mercator (UTM) coordinate system (EPSG:26916 and EPSG:26917). Raster data are stored as GeoTiff (.tif) files and vector data are stored as GeoPackage (.gpkg) files. The data are available for download via the USFS Research Data Archive [1] and the structure of the data are described below (Table 1).

**Table 1:** Structure and description of the sub-directories containing raster- and vector-based data.

*Main directory*	*Sub-directory*	*Content type*
*RDS-2024-0019_Metadata_Fileindex.zip*		*Variable descriptions* *File index*
*RDS-2024-0019_Supplements.zip*		*R code* *Lidar metadata*
*RDS-2024-0019_Data_BentCreek.zip*	*Grids*	*Raster data*
	*Vectors*	*Vector data*
*RDS-2024-0019_Data_Coweeta.zip*	*Grids*	*Raster data*
	*Vectors*	*Vector data*
*RDS-2024-0019_Data_Escambia.zip*	*Grids*	*Raster data*
	*Vectors*	*Vector data*
*RDS-2024-0019_Data_Hitchiti.zip*	*Grids*	*Raster data*
	*Vectors*	*Vector data*

The structure of the sub-directories containing raster data is described below (Table 2), using Bent Creek as an example.

**Table 2:** Description of the sub-directories containing raster-based data.

Filename	Variable	Units
aspect.tif	Aspect	degrees
canopy_cover.tif	Canopy cover	%
chm.tif	Canopy height model	meters
crr.tif	Crown relief ratio	unitless
dem.tif	Digital elevation model	meters
fhd.tif	Foliar height diversity	unitless
flowdir.tif	Flow direction	unitless
roughness.tif	Roughness	meters
slope.tif	Slope	degrees
top_rug.tif	Top rugosity	unitless
TPI.tif	Topographic position index	unitless
TRI.tif	Topographic roughness index	unitless
uci.tif	Understory complexity index	unitless
vci.tif	Vertical complexity index	unitless
vdr.tif	Vertical distribution ratio	unitless
z_max.tif	Maximum height of vegetation	meters
z_mean.tif	Mean height of vegetation	meters
z_sd.tif	Standard deviation of height	meters

Aspect refers to the orientation or azimuth of the terrain surface, measured clockwise in degrees from 0 to 360, where 0 is north-facing, 90 is east-facing, 180 is south-facing, and 270 is west-facing [3]. Canopy cover is the proportion of the ground surface area that is covered by the vertical projection of vegetation or tree canopy [4]. Canopy height model (CHM) is the height of tree canopies derived from the highest elevation of a ground-normalized point cloud [5]. Crown relief ratio (CRR) is the ratio of the vertical extent of a tree's crown to its total height [6]. Digital elevation model (DEM) is the terrain surface elevation above sea level [7]. Foliar height diversity (FHD) is the variation in vertical positions of foliage within a vegetation canopy, indicating the presence of different layers or heights of leaves and branches in a plant community [8]. Flow direction is the direction of the greatest drop in elevation, or the smallest rise if all neighbors are higher [9]. Roughness is the irregularity and variation in elevation across a DEM [10]. Slope is the steepness of the Earth's surface [3]. Top rugosity is the degree of irregularity or variation in the vertical profile of a vegetation canopy, reflecting the complexity and three-dimensional structure of the foliage distribution within the canopy layer [11]. Topographic position index (TPI) the difference between the value of a cell and the mean value of its 8 surrounding cells [10]. Topographic roughness index (TRI) is the mean of the absolute differences between the value of a cell and its 8 surrounding cells [10]. Understory complexity index (UCI) is the structural diversity and arrangement of vegetation at different vertical levels within a habitat, providing insights into the intricacy of the plant community's three-dimensional organization and is limited to lidar returns ≤3 m [12]. Vertical complexity index (VCI) is the structural diversity and arrangement of vegetation at different vertical levels within a habitat, providing insights into the intricacy of the plant community's three-dimensional organization [13]. Vertical distribution ratio (VDR) is the proportion of vegetation biomass or some other characteristic distributed within specific height intervals of a plant community, revealing the vertical arrangement of features such as leaves, branches, or vegetation density [14]. Z max is the maximum height of lidar returns within the grid cell. Z mean is the average height of lidar returns within the grid cell. Z sd is the standard deviation of lidar returns within the grid cell.

The structure of the vector data is described below (Table 3), using Bent Creek “crowns_dalponte_lmf.gpkg” file as an example. Each sub-directory contains a total of four files, and each file contains the same number of variables (*N* = 13). File names reflect the algorithms utilized in tree detection and crown delineation; for instance, "crowns_dalponte_lmf.gpkg" indicates that the tree detection step was performed via a manually defined local maximum filter (lmf) and the Dalponte algorithm [3] was used to segment the point cloud for crown delineation. Similarly, "crowns_silva_lmfauto.gpkg" denotes crown delineation with the Silva algorithm [15] and tree detection using the lidR_plugins package's lmfauto function [16].

**Table 3:** Description of the sub-directories containing vector-based data.

Filename	Variable	Units
crowns_dalponte_lmf.gpkg	treeID	unitless
	canopy_cover	%
	crr	unitless
	fhd	unitless
	top_rug	unitless
	uci	unitless
	vci	unitless
	vdr	unitless
	z_max	meters
	z_mean	meters
	z_sd	square meters
	crown_area	meters
	crown_diameter	meters

## 4 Experimental Design, Materials and Methods

### 4.1 Study area

**Fig. 1:**
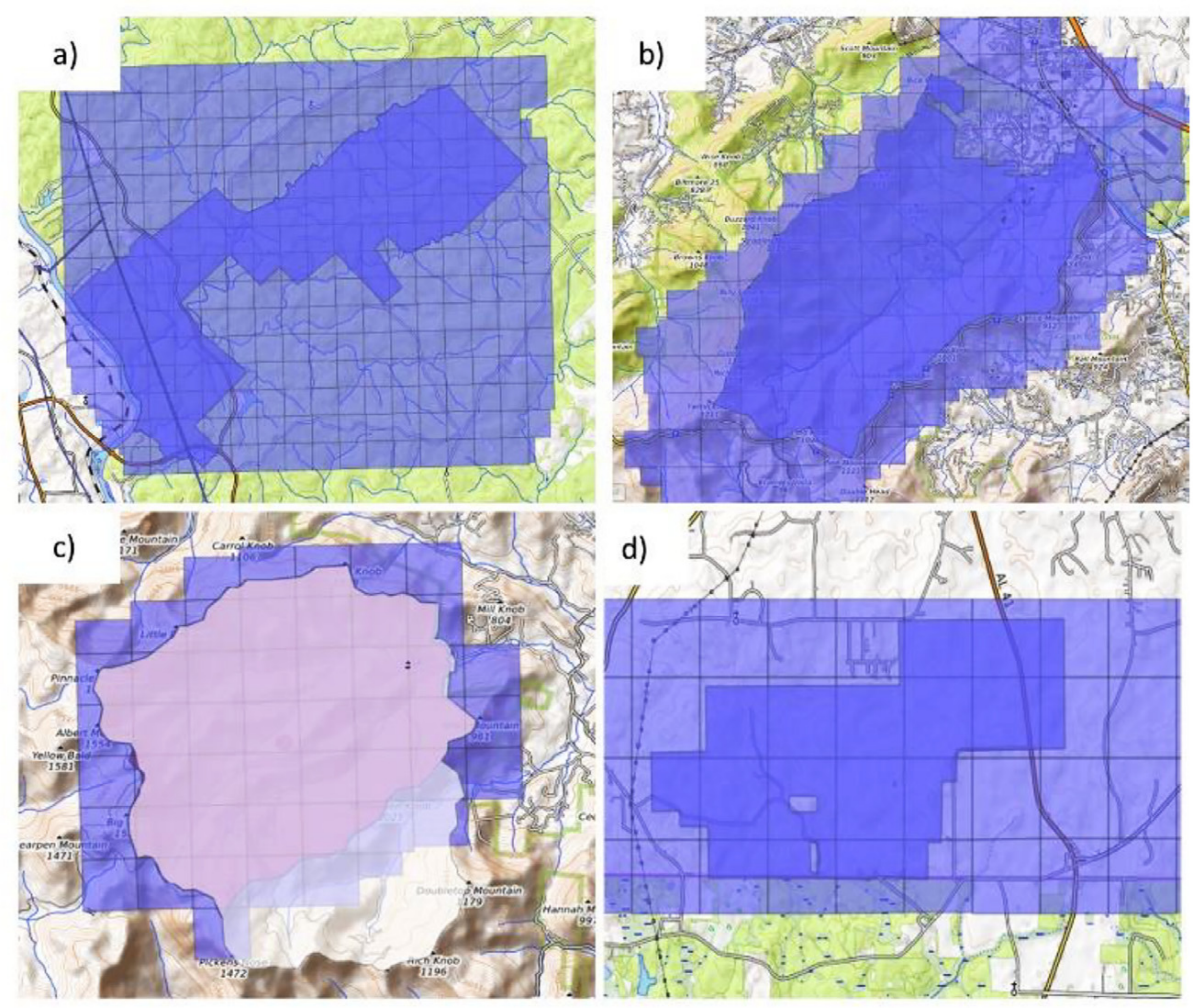
Aerial lidar coverage collected for a) Hitchiti, b) Bent creek, c) Coweeta, and d) Escambia experimental forests.

Lidar-derived data products were developed for four of the 84 US Forest Service's network of long-term Experimental Forests and Ranges (EFRs), including Bent Creek, Coweeta, Escambia, and Hitchiti. Established in 1908, the EFRs is the longest-running ecological research network in the US, providing an incredible wealth of records and knowledge regarding ecological change in natural and managed forest and grassland ecosystems. Individual sites range in size from 47 to 22,500 ha and are hosted on a combination of both public and private lands. Moreover, the network provides a home for long-term science and management studies in most of the dominant land cover types across the US (Fig. 1).

### 4.2 Lidar data collection and processing

ALS data acquisition for each EF was performed in the winter/early spring during the leaf-off period using an aircraft-mounted Optech Galaxy T2000 lidar sensor. Mean point density ranged from 42 to 74 points m^2^. Lidar data was provided by the vendor as laz files and geo georeferenced to the respective UTM (Universal Transverse Mercator) zone. Additional metadata is provided in Table 4. Data processing was divided into three primary phases, including 1) point-cloud cleaning and classification, 2) the generation of pixel-level metrics, and 3) tree-level metrics. All processing was performed in the R environment [17], primarily relying on package functions from lidR [5,7,16], tidyverse [[17], [18]], sf [19], and terra [9]. Processing was performed in parallel using the lidR las catalog functionality on a Windows PC with 256 GB of RAM and an AMD Ryzen Threadripper 3960 × 24-core processor.

**Table 4:** Lidar acquisition parameters.

Project name	Bent creek	Coweeta	Escambia	Hitchiti
Min/Max Ground Elevation	2000′−4000′	2200′–4900′	150′–250′	250′−625′
Flight Altitude	5600′	5000′	5400′	4000′
Distance Units	Meters	Meters	Meters	Meters
CRS	UTM 17N	UTM 17N	UTM 16N	UTM17N
Horizontal Datum	NAD83 - 2011	NAD83 - 2011	NAD83 - 2011	NAD83 - 2011
Vertical Datum	NAVD88–Geoid18	NAVD88–Geoid18	NAVD88–Geoid18	NAVD88–Geoid18
Scan FOV	30	30	30	18
Flying Altitude AMSL	8250′	8500′	5600′	1600′
Pulse Rate (kHz)	1500	1400	1500	585
Scan Rate (Hz)	110	100	110	61
Laser Power %	HIGH/STD	HIGH/STD	HIGH/STD	100
Swath Width	3000′	2700′	2800′	2100′
Planned Sidelap	30 %	30 %	30 %	30 %
Mean Point Density	15 points/m^2^	15 points/m^2^	15 points/m^2^	NA
Aircraft	Piper Navajo Chieftain	Piper Navajo Chieftain	Piper Navajo Chieftain	Piper Navajo Chieftain
Date Flown	12/15/21	11/23/21	11/02/21	01/04/21
Flight Speed	∼150 knots	∼150 knots	∼150 knots	
Base Station Type	Trimble RTX	Trimble RTX	Trimble RTX	Trimble RTX
GPS/INS Notes	POSpac v8.7	POSpac v8.7	POSpac v8.7	POSpac v8.7
Boresight Calibration	yes	yes	yes	yes
Minimum/Maximum Scan Angle Output	±15 degrees	±15 degrees	±15 degrees	±15 degrees
Actual Scan Angle Output	full FOV	full FOV	full FOV	full FOV
Tile Layout	750 m tiles	750 m tiles	750 m tiles	NA
Class 1	Unclassified	Unclassified	Unclassified	Unclassified
Class 2	Ground	Ground	Ground	NA
Class 5	Vegetation	Vegetation	Vegetation	NA
Class 6	Building	Building	Building	NA
Class 7	Low Points	Low Points	Low Points	NA
Data Formats Created	LAS v1.4	LAS v1.4	LAS v1.4	LAS v1.4
Processing Notes	minimal editing	minimal editing	minimal editing	NA
Number of Control Points Used in Analysis	3	2	5	8
Number of Control Points Eliminated from Original Set	0	0	0	2
Reasons for Point Removal	N/A	N/A	N/A	out of area
Average Elevation Variation	−0.001	−0.013	0.002	0.01
Minimum Elevation Dz	−0.003	−0.062	−0.052	−0.101
Maximum Elevation Dz	0.001	0..37	0.920	0.016
RMS	0.002	0.051	0.052	0.086
Horizontal Accuracy	<1 m	<1 m	<1 m	<1 m

### 4.3 Point-cloud classification and cleaning

Lidar data inherently includes the presence of noise and irrelevant data. This noise can originate from various sources, such as atmospheric disturbances, sensor anomalies, or reflections from unintended surfaces. Thus, point-cloud cleaning improves the overall quality of the lidar dataset and the resulting products. Classifying and cleaning lidar data reduces the volume of non-essential data, which is not only beneficial for streamlining data storage but also for minimizing the computational resources required for processing and analysis. This section describes the steps taken in order to clean the lidar point cloud.

**Fig. 2:**
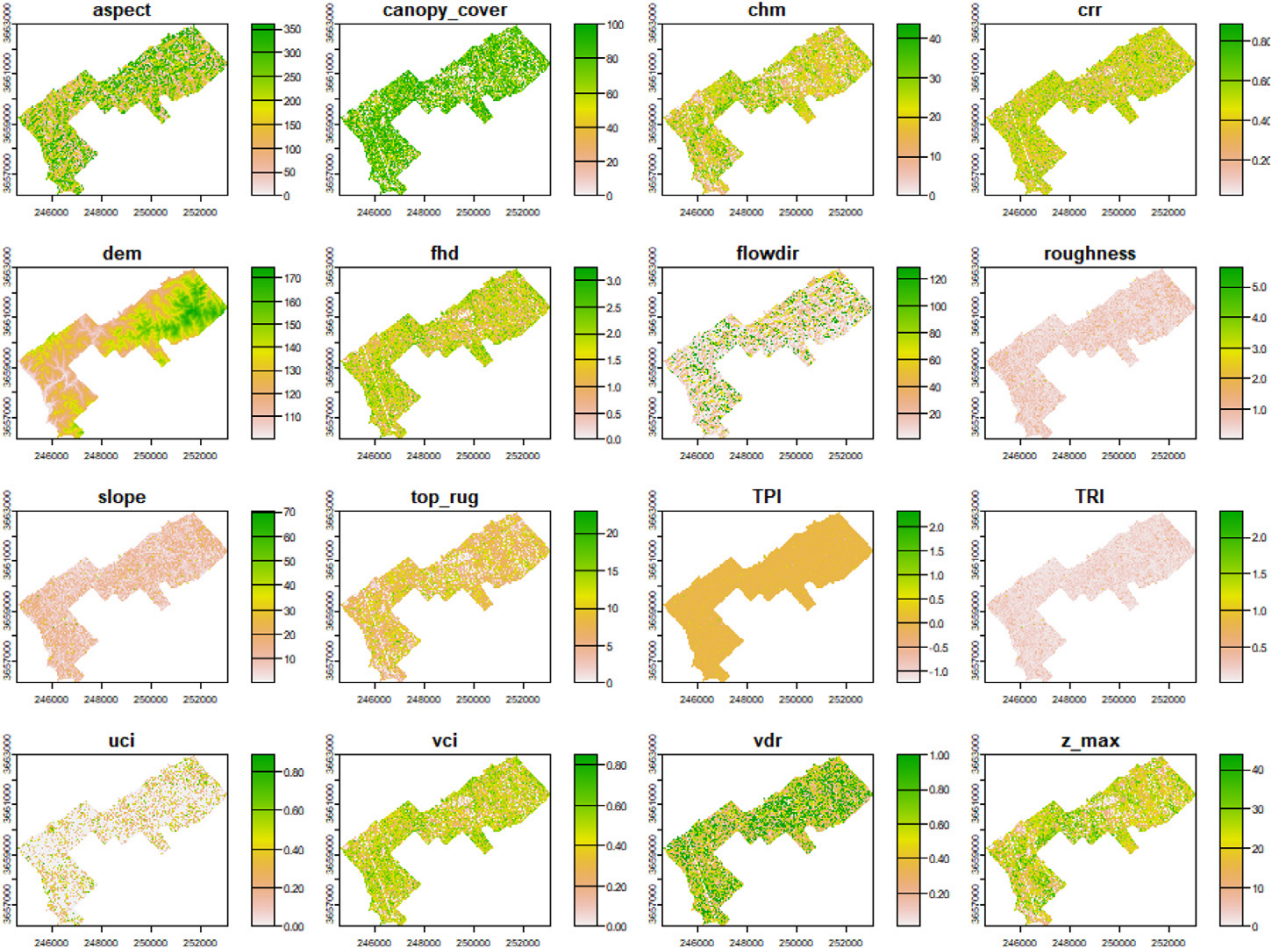
Example of forest structural complexity metrics for the Hitchiti Experimental Forest.

Point-cloud classification included identifying and classifying noise (i.e., outlier) as well as buildings and powerlines. The statistical outlier removal (SOR) algorithm available in the lidR classify_noise function was used to identify and segment (classify) noise in the point cloud. For each point, SOR computes the mean distance to all its k-nearest neighbours. Lidar returns that are farther than the average distance plus a number of times (multiplier) the standard deviation are considered noise. In some instances, it may be necessary to perform two or more ‘sweeps’ to classify and remove noise. Shapefiles representing building footprints and powerlines were used to identify and classify lidar returns to be removed from the workflow using the lidR classify_poi function. Shapefiles for building footprints and powerlines were queried from OpenStreetMap (OSM) [20] and merged into a single shapefile that was then used to segment the point cloud. For this analysis, buildings and power lines were classified as noise and removed. This was done to ensure that buildings and powerlines were omitted from the generation of pixel- and tree-level metrics (Fig. 2). Classification of ground returns was performed using the cloth simulation function [21]. Duplicate points (lidar returns with coincident *X, Y*, and *Z* values) and degenerated ground points (coincident *X* and *Y*) were also identified and removed.

### 4.4 Data products

Both pixel-level and tree-level products were computed from the aerial lidar data to capture a comprehensive representation of the forest structure [1]. The generation of pixel-level surface models, such as elevation and canopy height models, provides an overview of the landscape, while tree-level products, including individual tree detections and measurements, contribute to a finer-grained understanding of tree attributes. This dual approach aims to leverage the strengths of both pixel- and tree-level analyses for a more holistic assessment of the forest ecosystem. To alleviate edge effects, both pixel- and tree-level data products extend 200 m beyond the boundaries of the EF.

### 4.5 Pixel-level metrics

A digital elevation model (DEM) with spatial resolution of 1 m^2^ was generated from the cleaned, non-height-normalized point cloud using the lidR grid_terrain function on points classified as ground returns. The DEM was then used with the terra packages terrain function to generate additional topography-related surface models, including slope, aspect, topographic position index (TPI), terrain ruggedness index (TRI), roughness, and flow direction. A 3 × 3 moving window was applied to all gridded data products to fill in potential NA values before exporting as GeoTiff files. The point cloud was then height-normalized using the K-nearest neighbour algorithm with the lidR normalize_height function, which was then used to generate pixel-level metrics regarding forest structure at 1 m^2^ resolution using the lidR pixel_metrics function (Fig. 2). Forest structural metrics included canopy height (CHM), foliar height diversity (FHD), vertical distribution ratio (VDR), top rugosity (top_rug), crown relief ratio (CRR), understory complexity index (UCI), vertical complexity index (VCI), canopy cover, mean vegetation height (z_mean), max vegetation height (z_max), and standard deviation of vegetation height (z_sd).

### 4.6 Tree-level metrics

Derivation of tree-level metrics consisted of three primary steps: 1) individual tree detection (ITD), 2) individual tree segmentation (ITS), and individual crown delineation (ICD). Individual tree detection is the process of spatially locating trees and extracting height information. Individual tree segmentation is the process of segmenting (classifying) the point-cloud based on the trees detected in the previous step. Once the point cloud is segmented, the crowns of individual trees can be delineated, and tree-level metrics can be generated. Each of these three steps can be performed on a point-cloud or a canopy height model. Tree-level products were output using the open, non-proprietary, standards-based, and platform-independent GeoPackage format.

### 4.7 Individual tree detection

With the lidR package, trees are detected by applying a Local Maximum Filter (LMF) to either the point-cloud or a CHM. The processing of a point-cloud or a CHM is performed in a similar manner. For a given point (or pixel), the algorithm analyses neighbourhood points or pixels, checking if the processed point or pixel is the highest. A large window is preferable for tall trees while a small window size is preferable for short trees because crown size is correlated with tree height [22]. Because trees of variable sizes are often present in a single scene, a window size that adapts to tree height is preferable. For this analysis, individual tree detection was performed on the canopy height. model using two algorithms, both of which are based on the LMF approach using a variable window size with the lidR locate_trees function.

The window size of the first algorithm (lmf) was calculated by using a simple yet efficient linear relationship starting with a minimum window size of 3 m if tree height is less than or equal to 2 m, and gradually increases by 0.2 m for each incremental unit of tree height. However, to prevent the window size from exceeding 6 m, an upper limit is imposed when tree heights surpass 15 m. This modification ensures a balanced and controlled relationship between tree height and window size, as very large window sizes typically detect fewer trees.

The second algorithm, lmfauto, is still under development but is available with the lidRplugins package and implements a fast, parameter-free individual tree detection algorithm optimized for processing large areas efficiently [16]. It uses LMF in two steps or passes. The first pass performs a very rough estimation of the number of trees using a fixed window size. Based on the estimate from the first pass, it automatically computes a variable window size. According to the documentation (https://github.com/Jean-Romain/lidRplugins), this algorithm is suitable for processing large areas (e.g., forest scale) rather than small plots.

### 4.8 Individual tree segmentation

Individual tree segmentation using the lidR segment_trees function was performed on the point cloud using two algorithms: 1) silva2016 [15] and 2) dalponte2016 [23]. The silva2016 algorithm is based on seed and voronoi tessellation, which is similar to the nearest neighbour algorithm and requires a canopy height model and a set of individual seed points representing the tree locations. The Dalponte2016 algorithm is a seeds and growing region algorithm and requires a canopy height model and a set of individual seed points representing tree locations.

These algorithms were chosen based on results reported by Tatum and Wallin [24]. While each segmentation algorithm has its strengths and weaknesses, Tatum and Wallin [24] reported that Dalponte2016 performed best during data-model evaluation, followed closely by silva2016. Upon visual examination of the tree crowns, the authors reported that the crowns produced by the Silva2016 algorithm tended to be slightly larger and often over-extended into the non-canopy area (such as surrounding clearings, or inter-canopy gaps). Each ITS algorithm uses the tree locations from ITD to segment the point cloud and assign unique IDs to each return in the point cloud by inserting a new attribute named treeID in the LAS object header.

### 4.9 Individual crown delineation and tree metrics

The final step for derivation of tree-level metrics involves the delineation of tree crowns using the lidR crown_metrics function, which can be returned as a raster or a shapefile. For this analysis, crown delineation was performed on the point cloud using the crown metrics function, which simultaneously computes a set of user-defined metrics for each delineated tree crown. The same set of metrics derived at the pixel-level were calculated for each detected tree, in addition to crown area and mean crown diameter. Additionally, the generalized additive model (GAM) that was developed from the inventory data was used to predict bole height at the tree-level.

## Limitations

The size of the raw point-cloud data precluded storage on the USFS Research Data Archive. Therefore, this data is accessible for download from box.com (https://app.box.com/s/4s3412g8mtky0hb6wb63a44epv08c22o). Additionally, the volume of the point-cloud data may pose computational limitations.

## Ethics Statement

The authors have read and follow the ethical requirements for publication in Data in Brief and confirm that the current work does not involve human subjects, animal experiments, or any data collected from social media platforms.

## CRediT authorship contribution statement

**C. Wade Ross:** Conceptualization, Methodology, Software, Validation, Formal analysis, Investigation, Data curation, Writing – original draft, Writing – review & editing, Visualization, Project administration, Funding acquisition. **E. Louise Loudermilk:** Conceptualization, Methodology, Investigation, Writing – review & editing, Project administration, Funding acquisition. **Joseph J. O'Brien:** Conceptualization, Methodology, Investigation, Writing – review & editing, Project administration, Funding acquisition. **Grant Snitker:** Conceptualization, Methodology, Investigation, Writing – review & editing, Project administration, Funding acquisition.

## Data Availability

Forest structural-complexity metrics derived from aerial lidar across four experimental forests in the southeastern United States (Original data) (Research Data Archive).
